# Complementing but Not Replacing: Comparing the Impacts of GPT-4 and Native-Speaker Interaction on Chinese L2 Writing Outcomes

**DOI:** 10.3390/bs15040540

**Published:** 2025-04-17

**Authors:** Zhaoyang Shan, Zhangyuan Song, Xu Jiang, Wen Chen, Luyao Chen

**Affiliations:** 1 School of Foreign Languages and Literature, Shandong University, Jinan 250100, China; 202420267@mail.sdu.edu.cn; 2 School of International Chinese Language Education, Beijing Normal University, Beijing 100875, China; kristinasong@163.com (Z.S.); jx2927533599@163.com (X.J.); 3 Lishui Experimental School, Beijing Normal University, Lishui 323000, China; chenwen_hdqx@163.com; 4 Institute of Educational System Science, School of Systems Science, Beijing Normal University, Beijing 100875, China; 5 Department of Neuropsychology, Max Planck Institute for Human Cognitive and Brain Sciences, 04103 Leipzig, Germany

**Keywords:** second language writing, GPT-4, human language partner, large language models

## Abstract

This study explored the efficacy of large language models (LLMs), namely GPT-4, in supporting second language (L2) writing in comparison with interaction with a human language partner in the pre-writing phase. A within-subject behavioral experiment was conducted with 23 Chinese L2 learners who were exposed to three conditions: “without interaction”, “interaction with GPT-4”, and “interaction with a language partner”. They then completed an L2 writing task. It was found that interaction with the language partner yielded significantly improved results compared with both interaction with GPT-4 and the case without interaction in terms of overall writing scores, organization, and language. Additionally, both types of interaction enhanced the participants’ topic familiarity and writing confidence and reduced the task’s perceived difficulty compared with the case without interaction. Interestingly, in the “interaction with GPT-4” condition, topic familiarity was positively correlated with better writing outcomes, whereas in the “interaction with a language partner” condition, perceived difficulty was positively correlated with content scores; however, content scores were negatively associated with writing confidence. This study suggests that LLMs should be used to complement and not replace human language partners in the L2 pre-writing phase.

## 1. Introduction

The rapid advancement of artificial intelligence (AI) has led to the transformation of many fields, including second language acquisition (SLA) (Z. Zhang & Huang, 2024). Recent studies have demonstrated that AI tools—particularly large language models (LLMs), such as GPT-4—are reshaping language learning practices (Han, 2024; Lo et al., 2024). As the importance of effective second language (L2) writing skills is being increasingly recognized, pedagogical approaches that enhance writing performance through interaction and feedback are attracting interest (X. Zhang, 2023). In SLA, collaborative learning and peer feedback are widely documented as effective strategies that promote learner engagement and improve writing quality (Arques & Ferrero, 2023; Qian & Li, 2025). However, LLMs have opened up new opportunities for L2 writing as they can deliver human-like conversational responses (Austin et al., 2025) while providing contextually relevant feedback, mimicking the interactional dynamics found in peer collaboration (G. L. Liu et al., 2024; Naz & Robertson, 2024). Unlike traditional feedback mechanisms, which are often delayed and generic, LLMs can engage with learners in real time, offering tailored suggestions that align with the specific writing context (Dergaa et al., 2023).

Numerous studies have highlighted that LLMs such as GPT-4 can assist L2 learners in writing tasks by generating assessments (Tang et al., 2024), correcting grammatical errors (Guo, 2024), and enhancing vocabulary acquisition (Z. Zhang & Huang, 2024). In addition, the adaptive nature of LLMs allows for personalized learning experiences, potentially catering to individual learner needs more effectively than traditional methods (Ayeni et al., 2024; Jian, 2023). Thus, LLMs can not only provide diverse support for L2 writing but also empower learners to engage in more autonomous learning.

Despite these promising findings, it should be noted that the majority of existing studies have predominantly employed qualitative research methods to analyze the content of interactions and the writing feedback provided by LLMs (Tam, 2025). Studies have not yet quantitatively demonstrated how LLM-driven interventions compare to human interaction—especially with native speakers—in terms of their impacts on L2 learners’ writing performance. This gap in the literature is even more pronounced when considering the research participants. Existing research has focused on English as a foreign language (EFL) learners (Tsai et al., 2024), while studies examining Chinese L2 learners and their writing performance are scarce. Given the significant linguistic differences between Chinese and other languages, Chinese L2 learners often face distinct challenges, particularly associated with the inherent complexities of the Chinese script and syntax (Shu, 2024). As such, there is a critical need for more quantitative and experimental studies to investigate how LLMs could complement or even challenge traditional methods of language interaction and improve Chinese L2 learners’ writing.

This study intended to address this critical gap by quantitatively examining the effects of OpenAI’s GPT-4 on Chinese L2 learners’ writing performance through a within-subject behavioral experiment, thereby providing novel insights into Chinese L2 writing instruction. In particular, we aimed to understand how AI technologies such as GPT-4 can support and enhance L2 writing outcomes when compared with interaction with a human language partner. By critically evaluating the differences between AI-generated and human feedback, we aimed to provide guidance for pedagogical practices and contribute to a more comprehensive understanding of the ways in which innovative technologies can augment language education, particularly when teaching languages that are relatively challenging to learn as second languages.

## 2. Literature Review

### 2.1. Large Language Models in Language Learning and Teaching

LLMs are natural language processing technologies designed to represent and generate probabilities for vast amounts of text (M. Liu et al., 2023). Currently, various LLMs are available on the market, including ChatGPT (https://openai.com/index/chatgpt/, accessed on November 2022), which has become an indispensable tool for L2 teaching and learning (Kasneci et al., 2023). The generative LLM utilized in this study was GPT-4, the latest and most advanced version of the GPT series.

Research on the application of LLMs in language education has expanded significantly. Hong (2023) examined how learners and educators utilize GPT products, concluding that these tools could enhance language learning and assessment. Regarding learners, important insights were provided by Zheng et al. (2025), who demonstrated that GPT-4 significantly enhanced students’ oral proficiency during English learning. Additionally, Lelepary et al. (2023) focused on the assistance provided by GPT products among Arabic learners. They found that they helped to increase students’ reading skills and fostered a more interactive learning environment. Regarding educators, Jeon and Lee (2023) highlighted the roles of ChatGPT as an interlocutor, content provider, teaching assistant, and evaluator by generating contextual feedback, underscoring its potentiality in language teaching tasks. Nonetheless, several studies have pointed out that while LLMs could provide immediate feedback, the quality and contextual relevance of such feedback may vary significantly, potentially undermining these models’ effectiveness (Chang et al., 2024; L. Yan et al., 2024). This variability suggests that, despite their advantages, LLMs may not consistently address learners’ needs across different contexts. The studies by Alqahtani et al. (2023) and Da Silva et al. (2024) indicated that although LLMs could assist in language tasks, the lack of nuanced understanding in their feedback might limit their impacts on advanced learners. It is apparent that the application of LLMs in language education requires further in-depth exploration.

The application of LLMs in international Chinese education is another key area of increasing interest and scrutiny. Many scholars believe that ChatGPT holds great potential in international Chinese education and could serve as a powerful auxiliary tool for teachers (C. Chen & Gong, 2025). However, these technologies also present challenges (Deng, 2024; L. Gu, 2023). Specifically, Yuan and Wu (2023) discussed the opportunities, risks, and coping strategies associated with the use of GPT-4 in this field, emphasizing that people represent the core of Chinese education, while technology is merely an auxiliary tool. Deng (2024) demonstrated the necessity and feasibility of using ChatGPT as an auxiliary teaching tool in Chinese L2 learning, highlighting GPT-4’s multimodal features while acknowledging its limitations. Moreover, L. Gu (2023) examined the impacts of GPT products on the training of international Chinese teachers, proposing changes in the requirements, content, and methods of Chinese L2 learning. As a result, technologies such as GPT products cannot replace international Chinese teachers; rather, it is suggested that international Chinese education should transition from traditional models to a “human-centered, AI-supported” teaching approach.

Furthermore, it is necessary to examine the efficacy of LLMs in writing contexts. D. Yan (2023) highlighted how students engaged with GPT products in writing exercises, revealing insights into their perceptions and learning behaviors. Meanwhile, S. Wei and Li (2023) compared teacher feedback and AI-generated responses, analyzing the advantages of LLMs in terms of grammar correction and vocabulary enhancement. Similarly, G. Kurt and Y. Kurt (2024) investigated the use of ChatGPT as an automated feedback tool to assist EFL learners in writing. In addition, a series of studies have explored the overall impact of ChatGPT on students’ writing performance from a qualitative perspective (X. Li et al., 2023; Nguyen et al., 2024). While the existing research has demonstrated that LLMs can offer substantial support during or after the writing process, more targeted research is needed to explore their impacts on L2 learners in the early stages of writing instruction.

Notably, previous research on the use of LLMs such as GPT-4 in language learning and teaching has predominantly been qualitative or focused on literature reviews. Meanwhile, there is a lack of experimental evidence to substantiate their impacts on actual language learning outcomes, particularly in the context of Chinese L2 writing. Therefore, in this study, we explored the effects of LLMs, represented by GPT-4, in aiding Chinese L2 learners with pre-writing guidance and related aspects. Through such investigations, it is possible to appreciate the potential of tools such as GPT-4 to enhance the writing outcomes of L2 learners and inform pedagogical strategies that effectively blend traditional teaching methods with advanced AI capabilities.

### 2.2. Interactive Collaboration in L2 Writing

Interactive collaboration is an instructional approach that emphasizes active engagement and cooperation among learners and instructors (Qureshi et al., 2023), fostering a more dynamic learning environment. Rather than focusing on knowledge transmission, this approach encourages learners to construct an understanding through collaborative practices, such as peer feedback (Janesarvatan & Asoodar, 2024), joint writing tasks (Landrieu et al., 2024), and guided discussions (Zubiri-Esnaola et al., 2020), thereby enhancing their learning engagement and motivation (Miao et al., 2022).

There is growing interest in interactive collaboration within L2 learning, especially with regard to writing. Research has indicated that learners can deepen their understanding of language organization and use through dialog-based interactions, thereby improving both the accuracy and fluency of their L2 writing (Fernández-Dobao, 2020). Moreover, recent studies have highlighted not only the linguistic benefits of this approach but also the cognitive and social gains, such as increased learner autonomy (Chowdhury, 2021), confidence (Rahimi & Fathi, 2022), and critical thinking (Warsah et al., 2021), all of which are crucial for L2 writing development. Several studies have also noted the social dimensions of L2 writing, emphasizing the importance of interpersonal relationships and social interactions in the L2 writing process (Q. Li & He, 2017; Wen & Zhang, 2022). Specifically, a study conducted by Wen and Zhang (2022) revealed that fostering positive social dynamics in collaborative settings can enhance learners’ motivation and engagement in L2 writing. This focus on the social aspects of L2 writing underscores the need for a supportive community in which learners can thrive.

It is worth noting that with the advancement of LLMs, significant transformations have been observed regarding interactive collaboration in L2 writing, as LLMs offer new avenues for enhanced interaction (Tabone & De Winter, 2023), feedback (G. L. Liu et al., 2024), and collaborative knowledge construction (Su et al., 2023). For instance, several studies have examined the use of LLMs in collaborative writing tasks, showing that such tools can facilitate peer feedback and improve overall writing quality (Lingard, 2023; Zuckerman et al., 2023). This indicates a shift toward hybrid learning environments that blend face-to-face interactions with digital collaboration.

While existing research highlights the effectiveness of interactive collaboration in enhancing L2 writing (Fernández-Dobao, 2020), there is a noticeable gap in the literature concerning interactions that occur before the actual writing phase. Previous studies have predominantly focused on how peer feedback or teacher feedback during the writing process contributes to improved subsequent writing quality (Y. Fan & Xu, 2020; D. Li & Zhang, 2022). However, limited attention has been given to the preparatory stage, where learners engage in collaborative discussions, brainstorming, and idea sharing before drafting their written work. This stage, which is often overlooked, plays a critical role in reducing the cognitive load and improving the quality of students’ writing (Q. Y. Gu & Jin, 2021). In addition, few studies have explored the application of LLMs in the pre-writing phase and their impact on writing. Therefore, the goal of this study was to investigate the impact of pre-writing interactions with LLMs, exemplified by GPT-4, and with a native language partner on the writing outcomes of Chinese L2 learners. In this way, we sought to determine how LLMs can complement traditional collaborative methods to enhance Chinese L2 learning.

### 2.3. Topic Familiarity, Writing Confidence, and Perceived Difficulty in L2 Writing

Topic familiarity refers to whether students write about common everyday topics with which they are familiar (+familiar) or topics with which they are unfamiliar (−familiar) (Yang & Kim, 2020). According to Robinson’s (2011) framework of cognitive task complexity, topic familiarity can divert L2 learners’ attention resources away from language use or development. A number of studies have demonstrated that topic familiarity has a significant impact on the quality of written text, as well as on CAF measures (e.g., lexical complexity, accuracy, fluency, etc.) (Kessler et al., 2022). For instance, Abdi Tabari et al. (2024) found that writing differed systematically in terms of linguistic complexity due to the influence of topic familiarity, and familiar topics led to writing with a higher level of linguistic complexity as compared with unfamiliar topics. Through a comparison study, Bui and Luo (2021) found that young learners who wrote about familiar topics (experimental group) produced longer texts and demonstrated greater lexical diversity compared with those who wrote about unfamiliar topics (control group). As a result, topic familiarity appears to play a critical role in L2 writing.

Writing confidence is equally crucial in L2 writing. Bandura (1986) proposed that individuals’ beliefs in their abilities (self-efficacy) significantly influence their behavioral patterns. In other words, individuals with high self-efficacy are often confident in their abilities to complete a particular task and thus anticipate successful outcomes, whereas those with low self-efficacy are more likely to anticipate failure. Specifically, in the context of L2 writing, writing confidence can be regarded as a concrete manifestation of one’s self-efficacy in writing situations, with the two being highly consistent in essence (Pajares & Johnson, 1994). Due to limitations in language proficiency, learners often face greater cognitive challenges; thus, writing confidence is one of the key factors influencing their writing performance (Golparvar & Khafi, 2021). Relevant studies have shown that self-efficacy beliefs regarding writing (i.e., writing confidence) are closely related to writing performance (Mitchell et al., 2023). Learners with high levels of writing confidence not only demonstrate greater accuracy in language use but also organize content and express their ideas more effectively (Prat-Sala & Redford, 2012). Conversely, learners with low levels of writing confidence are more prone to self-doubt, which can hinder their ability to produce coherent and sophisticated texts (Busse et al., 2023). A meta-analysis also found that the writing confidence of English learners (i.e., English writing self-efficacy) had a significant impact on their English writing performance (Sun et al., 2021).

Furthermore, multiple studies have pointed to perceived difficulty as an important factor in L2 writing (C. Fan & Wang, 2024; X. Wei et al., 2020). Perceived difficulty refers to whether individuals perceive a certain behavior as difficult to perform (Trafimow et al., 2002). In the context of L2 writing, perceived difficulty is a subjective judgment that reflects learners’ assessment of the cognitive and linguistic demands of a writing task (Sasayama, 2016). This perception is influenced by factors such as the complexity of the task, the learner’s language proficiency, and their skill level (Cho, 2018). Research has indicated that when learners perceive writing tasks as highly difficult, they are more likely to experience cognitive overload and emotional stress, which can negatively impact their writing performance (Malagoli et al., 2021). This phenomenon is particularly pronounced among EFL learners, who often face additional challenges due to the linguistic and cultural differences between their native language and English (Malt & Sloman, 2003). In particular, a study based on the fsQCA approach found that EFL learners who exhibited high levels of perceived writing difficulty were more likely to experience a decline in writing performance (C. Fan & Wang, 2024).

The integration of LLMs into language learning has transformed learners’ topic familiarity, writing confidence, and perceived difficulty, as they can offer learners personalized, adaptive, and non-judgmental interactions (G. L. Liu et al., 2024; Albdrani & Al-Shargabi, 2023). In the context of L2 writing, several studies have shown that learners who interact with LLMs report greater confidence in their abilities, suggesting that LLMs can serve as a valuable resource for improved writing performance (Bouzar et al., 2024; Kang & Pyo, 2024). Therefore, LLMs may significantly influence L2 writers’ topic familiarity and the perceived difficulty of writing tasks. While LLMs offer valuable personalized feedback, human interaction remains crucial in providing a strong social context (J. Chen et al., 2024). Thus, it is crucial to explore the impacts of interaction with LLMs versus interaction with human language partners on topic familiarity, writing confidence, and perceived difficulty in L2 writing students.

While some research has explored the impacts of topic familiarity, writing confidence, and perceived difficulty in L2 writing, few studies have focused on how LLMs such as ChatGPT influence these aspects. In this study, we designed an interactive questionnaire to collect data on topic familiarity, writing confidence, and perceived difficulty from Chinese L2 learners after interacting with GPT-4 in the pre-writing phase, with the aim of analyzing GPT-4’s potential in L2 writing tasks.

## 3. Methodology

### 3.1. Overview

This study sought to compare the efficacy of GPT-4 to that of a native Chinese language partner in supporting L2 writing performance. A within-subject behavioral experiment was designed to control for individual differences among the experimental conditions (see below). Chinese L2 participants completed writing tasks under three conditions during the 10 min pre-writing phase: without interaction (“W”), interaction with GPT-4 (“G”), and interaction with a native Chinese language partner (“P”). Based on the participants’ writing scores, we assessed and compared the effects of GPT-4 and the native Chinese language partner in supporting L2 writing.

In addition, we designed two 5-point interactive Likert scales (GPT Interaction Questionnaire and Peer Interaction Questionnaire) in this experiment to better evaluate the relationship between participants’ experiences in interacting with GPT-4 or the language partner and their L2 writing outcomes. Notably, participants in the “without interaction” condition were also required to independently think about the writing topic for ten minutes during the pre-writing stage and then complete the questionnaire to rate their topic familiarity, writing confidence, and the perceived difficulty of the task.

### 3.2. Research Questions

Q1: How do the overall writing score and its sub-dimensions (i.e., content, organization, language, and vocabulary) differ under different interaction conditions (GPT-4, human language partner, and without interaction)?

H1: Drawing on empirical evidence that interactive collaboration (peer-to-peer and teacher–student interactions) positively impacts L2 writing by enhancing accuracy (Fernández-Dobao, 2020) and fostering learner autonomy (Chowdhury, 2021), while LLMs have demonstrated their capacity to improve collaborative writing outcomes by providing contextually relevant feedback (G. Kurt & Y. Kurt, 2024) and optimizing interaction quality (Lingard, 2023), we hypothesize that L2 learners’ writing scores will be higher under interactive conditions compared with without interaction, with the scores from interaction with GPT-4 and human language partner being comparable, and potential variations across its sub-dimensions.

Q2: What are the relationships between writing outcomes and factors such as topic familiarity, writing confidence, and perceived difficulty under the three interaction conditions (GPT-4, human language partner, and without interaction)?

H2: Building on prior research indicating that individual learner factors such as topic familiarity (Kessler et al., 2022), writing confidence (Golparvar & Khafi, 2021), and perceived difficulty (C. Fan & Wang, 2024) are significantly related to L2 writing performance, and recognizing that these factors may interact differently with varying forms of writing support including AI-mediated and human-mediated feedback (J. Chen et al., 2024; G. L. Liu et al., 2024), we hypothesize that under different interaction conditions, writing outcomes will be differentially associated with learners’ topic familiarity, writing confidence, and perceived difficulty in L2 writing.

### 3.3. Methods

The independent variable was the type of interaction partner, i.e., whether the L2 writing task was supported by GPT-4 or a native Chinese language partner. The “without interaction” condition was set as the experimental baseline. Regarding the selection of the native language partner, the chosen individual was a graduate student majoring in Teaching Chinese as an International Language. He/She had prior experience as a language partner for Chinese L2 learners and was familiar with the three selected topics. These topics were of comparable difficulty, ensuring a consistent level of topic familiarity and reducing the potential for variability in the interaction experiences of the participants. The dependent variable was the L2 writing output/score under the three conditions: without interaction, with the assistance of GPT-4, or with the assistance of the native Chinese language partner. Furthermore, questionnaires were utilized to examine the participants’ topic familiarity and writing confidence and the task’s perceived difficulty under the three conditions, with the scores also serving as the dependent variables.

### 3.4. Participants

We recruited 30 Chinese L2 learners and asked them to complete a background questionnaire as a screening tool before the experiment in order to gather information about their demographic backgrounds, Chinese language proficiency, writing habits, etc. The inclusion criteria were as follows: participants were required to have a Hanyu Shuiping Kaoshi (HSK, i.e., Chinese Proficiency Test) level of 4 or above and at least three years of Chinese language learning experience, ensuring their familiarity with Chinese. Additionally, they were required to have prior experience in writing in Chinese.

Each participant was required to complete three writing tasks in full, with no arbitrary withdrawals allowed during the experiment. The participants signed a consent form and received remuneration after completing the experiment. This behavioral study was approved by the Ethics Committee of Beijing Normal University.

The final data analysis included data from 23 participants, as those from 7 participants were excluded due to factors such as dropout, insufficient Chinese language proficiency, and a lack of experience in Chinese writing. The remaining 23 participants had Chinese language proficiency levels ranging from HSK 4 to HSK 6, indicating that they were intermediate to advanced Chinese L2 learners, and they had a mean Chinese learning age of 16.4 years (*SD* = 2.8 years). They consisted of both undergraduate and graduate students, with a mean age of 24.6 years (*SD* = 4.9 years), and they spoke a variety of native languages, including Malay, Korean, Spanish, Mongolian, and others. Thus, they exhibited broad linguistic and cultural backgrounds. This diversity aligned with several influential L2 writing studies, such as those of Cumming (2001) and Haneda (2005), which emphasize the importance of including participants with diverse linguistic backgrounds in order to better understand the complexities of L2 writing. However, due to the limited sample size, we did not take the language background as an independent variable in this study, but it might be interesting for future larger-sample studies to explore how different language backgrounds affect L2 writing performance after interactions with LLMs and language partners.

### 3.5. Materials and Procedures

Regarding the selection of essay topics, three moderately challenging and comparable topics were chosen from the HSK Dynamic Composition Corpus at Beijing Language and Culture University. The topic difficulty was evaluated at two levels: firstly, by calculating the average scores for all responses for the corresponding topics within the HSK corpus, and secondly, through difficulty ratings provided by 40 native Chinese speakers. Based on these two analyses, three topics with similar average scores were selected. Specifically, the average scores for the three topics were 67.19 (*SD* = 8.3), 67.92 (*SD* = 7.9), and 68.43 (*SD* = 8.7), respectively. The median scores for these topics were 74, 73, and 75. These statistical findings confirmed that the three topics had similar levels of difficulty, as indicated by both the HSK corpus and the native Chinese speaker ratings. The topics were as follows: Topic A—*green food and hunger*; Topic B—*how to solve the generation gap problem*; and Topic C—*how to face setbacks*. The design of the scales in this study was informed by the studies of Bruning et al. (2013), Schmidt-Rinehart (1994), and Wu (2003), which guided the exploration of the learners’ topic familiarity, writing confidence, and perceived difficulty across the different interaction conditions.

Before the experiment, the researchers provided the participants with detailed instructions and training to ensure that they understood the task’s requirements. The participants practiced interacting with GPT-4 and the native Chinese language partner in the corresponding sessions according to the interaction protocols. The researchers did not instruct the participants on how to write the essays in terms of content.

**Figure 1:**
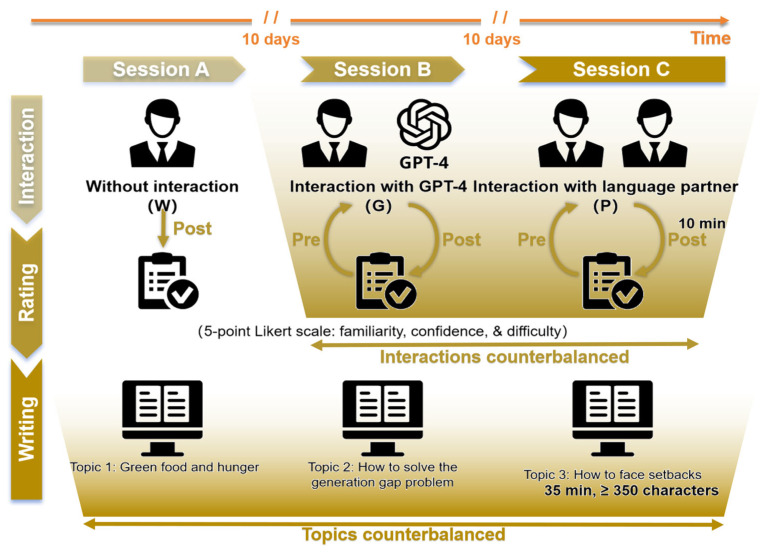
The experimental procedure. Each participant was invited to participate in a three-session writing experiment. Between each session, there was a minimum 10-day interval. In order to avoid carryover effects from the interactions, all participants first underwent a session involving writing without interaction; subsequently, the two interaction conditions (i.e., interaction with GPT-4 and interaction with the language partner) were counterbalanced across the participants. The writing topics were also counterbalanced across the three writing sessions.

During the experiment, the participants first completed a writing task without any interaction; they then alternated between interacting either with GPT-4 or with a native Chinese language partner prior to completing another two writing tasks. Each interactive condition was separated by a minimum interval of 10 days to reduce carryover effects. Additionally, the interactions and corresponding topics were counterbalanced among the participants. For each writing task, they were required to compose a response consisting of at least 350 Chinese characters within a 35 min time frame. Interaction with GPT-4 or the native language partner and self-completion without interaction lasted 10 min and took place immediately before the writing task. The participants were not permitted to interact with either GPT-4 or the native language partner during or after the writing task. The whole experimental procedure is shown in Figure 1.

During the pre-writing phase with interaction with either GPT-4 or the language partner, the participants were free to ask questions without restrictions. This was implemented based on the following rationale. Firstly, we adopted a reverse restriction approach by imposing limitations on both GPT-4 and the Chinese language partner. When the participants asked about specific writing content during their interactions, neither GPT-4 nor the language partner could provide sufficient amounts of text that the participants could directly copy for their essays. Secondly, in this study, we emphasized natural interaction. In real-life situations where a language partner assists a Chinese L2 learner with writing, there are typically no strict limitations on the questions that can be raised by the Chinese L2 learner. Thirdly, this study included interaction scales for both GPT-4 and the language partner, which were designed to track the participants’ experiences during their interactions with GPT-4 and the language partner. Lastly, since we adopted a within-subject design, individual variance in the interaction features was minimized between the comparisons, thus guaranteeing coherence among the different conditions.

To prevent the participants from directly copying content provided by GPT-4 or the native Chinese language partner, initial prompt settings were implemented in GPT-4. It was configured to provide topic-related knowledge, cultural information, and suggestions on writing organization, rather than generating a complete essay or paragraphs of content. Moreover, it only offered fragmented guidance, conceptual explanations, and thematic suggestions to inspire the participants, rather than supplying ready-to-use content. The participants were informed of these limitations, and regular monitoring was conducted to ensure adherence to the guidelines, effectively reducing the risk of direct copying. The same restrictions were applied to the native Chinese language partner to maintain consistency in the experiment. Furthermore, in order to prevent non-language information such as gestures, facial expressions, or eye contact from interfering with the interaction process, the language partner was required to turn off the video camera and to communicate with the participants solely through text, as in the case of GPT-4. The requirements set for GPT-4, as well as for the language partner in this study, were as follows:

“Please play the role of a Chinese language partner and help the international student you are conversing with to complete a 350-character argumentative essay. Before starting the conversation, you need to ask about their Chinese proficiency level and the essay topic that they need to write about. During the conversation, you should not provide a reference essay directly but instead assist them by asking questions and offering prompts to help them gradually develop and refine their essay. At the same time, you should pay attention to the accuracy of their Chinese language expression. Ensure that each exchange feels like a real conversation, guiding them step by step through the writing process.”

### 3.6. L2 Writing Assessment Criteria

This study followed the assessment rubrics created by Jacobs et al. (1981), which evaluate students’ writing according to five factors: content, organization, language, vocabulary, and mechanics. The dimensions are thus defined as follows: content refers to the relevance and depth of the ideas presented; organization reflects the clarity and logical flow of the essay; language refers to grammar and sentence construction; vocabulary focuses on the appropriateness and diversity of words; and mechanics pertains to spelling, punctuation, and formatting. We did not include the mechanics dimension in this study, as the essays were primarily in electronic format. Thus, only four dimensions were used to evaluate the scores of the essays in this study: content (30%), organization (20%), language (25%), and vocabulary (25%). The equal weighting between language and vocabulary reflected the importance of both syntactic fluency and vocabulary richness in assessing writing quality (Truckenmiller et al., 2021), especially in digital environments, where linguistic precision is paramount.

LLMs have been utilized in automated essay scoring systems, where deep learning techniques have been employed to understand the semantics of the text, analyze the grammar, and evaluate its organization, thereby improving the scoring accuracy (Song et al., 2024; Pack et al., 2024). Thus, in this study, considering the need for fairness and consistency in assessing the writing scores, a third party was selected to evaluate the participants’ Chinese L2 writing. This was another LLM based on Chinese corpora, namely ERNIE Bot (https://yiyan.baidu.com/, accessed on March 2023). ERNIE Bot, developed by Baidu, possesses the advantage of achieving a comprehensive semantic understanding of Chinese texts and the precise capture of linguistic features (X. Wei, 2024). Compared with other models that rely on English corpora (such as GPT), ERNIE Bot demonstrates superior contextual understanding and lexical analysis capabilities when assessing Chinese essays (Lin, 2024). Consequently, in this study, we employed ERNIE Bot to conduct automated scoring on the collected essays, using the four scoring dimensions described above: content, organization, language, and vocabulary. To guarantee both accuracy and fairness in scoring, we also referenced the official guidelines for essay scoring stipulated in the HSK exam documentation. This places a strong emphasis on the coherence and depth of writing, requiring examinees to demonstrate appropriate grammatical structures and a rich vocabulary in their language use (i.e., language expression) while also focusing on the logical flow and organization of the essay. During the scoring process, the following instructions were provided to ERNIE Bot:

“Please take on the role of an expert in Chinese essay evaluation, following the HSK exam’s scoring standards to assess the students’ essays. Begin with an overall analysis of the submitted text and then evaluate it across four dimensions: content, organization, language, and vocabulary. During the evaluation, pay special attention to the essay’s thematic coherence, grammatical accuracy, and lexical diversity. Instead of directly assigning a score, provide constructive feedback by identifying issues and offering suggestions. Ensure that the assessment process strictly adheres to the HSK scoring criteria to maintain fairness and consistency.”

Furthermore, three experienced Chinese language teachers (mean age = 30.6 years, *SD* = 1.88 years) were invited to evaluate the reliability of ERNIE Bot’s essay scoring, all with over four years of Chinese teaching experience. Nine essays scored by ERNIE Bot were randomly selected, with three essays per condition, and the scoring feedback was collected. In particular, for each essay, ERNIE Bot provided explanations for the scores assigned regarding the four dimensions (i.e., content, organization, vocabulary, and language). Subsequently, the three teachers were provided with the same grading criteria as given to ERNIE Bot. After familiarizing themselves with the essays’ contents and the scoring feedback from ERNIE Bot, the teachers independently rated their agreement with ERNIE Bot’s scores across the four mentioned dimensions through a 5-point Likert scale. The results reveal that the teachers’ agreement with ERNIE Bot’s scores ranged from “moderately agree” to “strongly agree”, thus demonstrating the reliability of ERNIE Bot’s scoring. Examples of ERNIE Bot’s scoring feedback, along with the questionnaire used to assess the teachers’ agreement with ERNIE Bot’s scoring and the results, are presented in the Supplementary Materials.

### 3.7. Data Analyses

The scoring procedure was repeated three times for each essay, simulating the assessment process implemented by three (human) L2 teaching experts. Kendall’s coefficient of concordance was calculated to ensure consistency in the grading process via the ERNIE Bot. If the scoring was highly consistent, the final essay scores for each student were determined by averaging the scores of the three assessments.

As several scores were not normally distributed (see Supplementary Materials for details), the non-parametric Friedman test was performed to identify the differences among the three interaction conditions regarding the total scores and the scores of the four writing evaluation dimensions (i.e., content, organization, language, and vocabulary), as well as for the ratings of familiarity, confidence, and difficulty. Wilcoxon’s signed-rank test with the continuity correction was employed for the corresponding post hoc comparisons if the Friedman test results were significant. Moreover, Spearman’s correlation tests were performed to evaluate the relationships between the writing and rating scores for the conditions, denoted as G (i.e., interaction with GPT-4) and P (i.e., interaction with the language partner), respectively. All data analyses were performed using R (version 4.3.0, https://www.R-project.org).

Furthermore, a complementary thematic analysis of the qualitative data (Braun & Clarke, 2006) obtained from the interaction transcripts (i.e., interactions with both GPT-4 and the language partner) was conducted to explore the differences between them, which might have influenced the writing outcomes under the different interactive conditions. The data were exported into a coding template using qualitative data analysis software (ATLAS.ti 22). An initial coding and sub-coding cycle was conducted to organize the data into themes (Miles et al., 2014). The data were further condensed through several rounds of observation and reflection, ultimately distilling the differences among the interaction conditions into five thematic categories, which are summarized in Appendix A. In this way, we sought to account for the differences in the writing outcomes in a complementary manner.

## 4. Results

### 4.1. Consistency in Writing Scores

The determination of Kendall’s coefficient of concordance yielded a coefficient value of 0.563, with a chi-squared value (*χ*^2^) of 77.673 and a significance level of *p* < 0.001. This result indicates that there was a relatively high level of concordance among the three rounds of scoring, demonstrating that the scores assigned were consistent and reliable across each evaluation. This confirmed the validity of the final averaged score as a fair representation of the participants’ L2 writing performance.

### 4.2. Differences in Writing Scores

**Figure 2:**
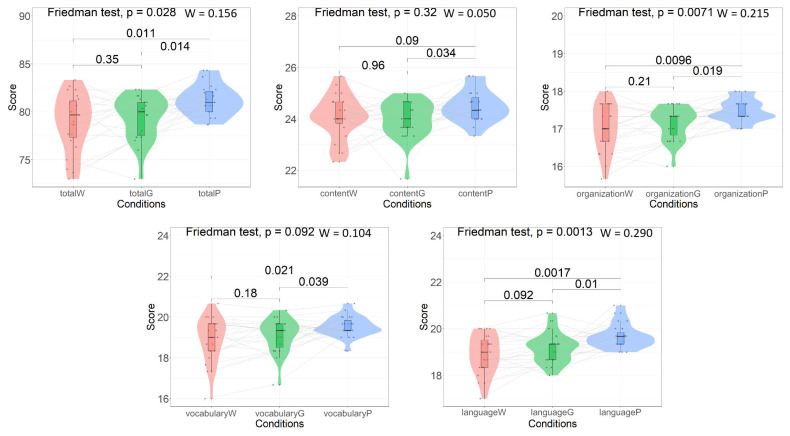
Writing score comparison results. *p*-values for the Friedman test with effect sizes (*W* denotes Kendall’s W) and *p*-values for the Wilcoxon signed-rank test are provided. On the horizontal axis, W—without interaction; G—interaction with GPT-4; and P—interaction with language partner.

The results of the Friedman test and Wilcoxon signed-rank test for the writing scores are summarized in Figure 2. The effect sizes for the Friedman test and Wilcoxon signed-rank test, as well as Kendall’s W (noted as “*W*” in the results), are also reported. It is noteworthy that for the Wilcoxon signed-rank test, the effect size—or Cohen’s r—was infinite because of the relatively small sample size. Thus, we adopted the absolute value of Cohen’s d (noted as “*d*” hereafter) as an alternative to enable a coarse evaluation.

The interaction conditions showed a significant difference regarding the total writing scores (*χ*^2^ = 7.167, *df* = 2, *p* = 0.028, *W* = 0.156). In particular, interaction with the language partner yielded superior results to the other two conditions (*Vs* ≥ 42, *ps* ≤ 0.014, *ds* ≥ 0.57). As for the four writing scores, both organization and language showed significant differences among the interaction conditions (*χ*^2^*s* ≥ 9.887, *df* = 2, *ps* ≤ 0.007, *Ws* ≥ 0.215). Similarly, interaction with the language partner led to higher scores than the other two conditions (*Vs* ≥ 16.5, *ps* ≤ 0.019, *ds* ≥ 0.60). Nevertheless, the language score for the “interaction with GPT-4” condition was marginally higher than that of the “without interaction” condition (*V* = 40.5, *p* = 0.092, *d* = 0.45). The vocabulary scores also showed differences across the interaction conditions (*χ*^2^ = 4.781, *df* = 2, *p* = 0.092, *W* = 0.104). No significant difference could be detected for the content scores (*χ*^2^ = 2.296, *df* = 2, *p* = 0.317, *W* = 0.050).

### 4.3. Differences in the Rating Scores

**Figure 3:**
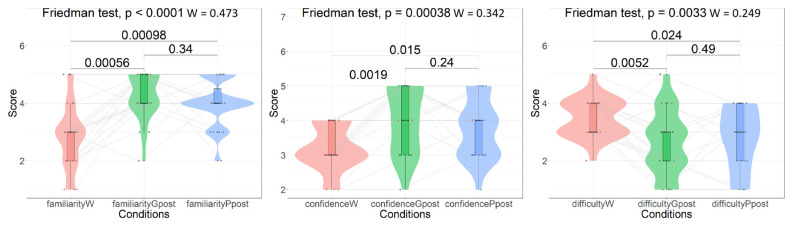
Rating score comparison results. *p*-values for the Friedman test with effect sizes (*W* denotes Kendall’s W) and *p*-values for the Wilcoxon signed-rank test are provided.

The results of the Friedman test and Wilcoxon signed-rank test with continuity correction for the rating scores are summarized in Figure 3. As previously mentioned, effect sizes are also reported.

All rating dimensions showed significant differences across the three interaction conditions (*χ*^2^*s* ≥ 11.446, *df* = 2, *ps* ≤ 0.003, *Ws* ≥ 0.249). Compared with the “without interaction” condition, both interaction with GPT-4 and interaction with a language partner were associated with significantly higher topic familiarity and writing confidence and lower levels of writing difficulty (*Vs* ≥ 10, *ps* ≤ 0.024, *ds* ≥ 0.74). Meanwhile, no significant differences could be found between the “interaction with GPT 4.0” and “interaction with a language partner” conditions (*Vs* ≤ 123.5, *ps* > 0.24) regarding familiarity (*p* = 0.34, *d* = 0.24), confidence (*p* = 0.24, *d* = 0.27), or difficulty (*p* = 0.49, *d* = 0.16).

### 4.4. Correlations Between Writing and Rating Scores

**Figure 4:**
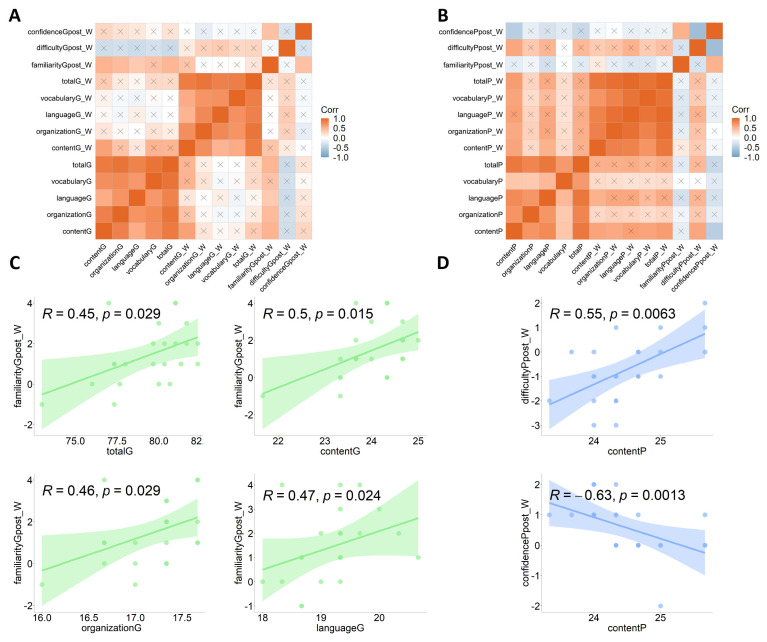
Spearman’s correlation test results. Spearman’s R values, as well as *p*-values, are provided. Post_W: rating score change, i.e., post-interaction rating score−rating score under the “without interaction” condition; G_W: writing score change, i.e., G = writing score under interaction with GPT-4−writing score under the “without interaction” condition; and P_W: writing score change, i.e., P = writing score under interaction with a language partner−writing score under the “without interaction” condition. (**A**) Heat map of the correlation between interaction with GPT-4 ratings (familiarity, confidence, and difficulty) and writing scores (total & sub-dimensions). (**B**) Heat map of the correlation between interaction with language partner ratings (familiarity, confidence, and difficulty) and writing scores (total & sub-dimensions). (**C**) Significant correlation between Gpost_W familiarity and writing scores (total & sub-dimensions). (**D**) Significant correlation between Ppost_W difficulty/confidence and content score.

As shown in Figure 4A,C, a change in the familiarity score as a result of interaction with GPT-4 (i.e., post-interaction familiarity rating score—familiarity rating score of the condition of “without interaction”) was positively correlated with the total writing score, as well as the content, organization, and language scores (Spearman’s *Rs* ≥ 0.45, *ps* ≤ 0.029).

Regarding interaction with the language partner, a change in the familiarity score was positively correlated with the content score (Spearman’s *R* = 0.55, *p* = 0.006), while a change in the confidence score was negatively correlated with the content score (Spearman’s *R* = −0.63, *p* = 0.001) (see Figure 4B,D).

## 5. Discussion 

In this study, we sought to compare the writing score differences in various interactive scenarios in the pre-writing phase and to evaluate the potential for improved writing scores via interaction with GPT-4. Our results show that during preparation, interaction with a language partner outperformed both the interaction with GPT-4 and the “without interaction” condition in terms of improving the total writing score, as well as the organization and language expression scores. Nevertheless, compared with the “no interaction” condition, both interaction with GPT-4 and interaction with a language partner significantly enhanced the participants’ topic familiarity and writing confidence and reduced the perceived level of writing difficulty in a similar pattern. Interestingly, a change in topic familiarity caused by the interaction with GPT-4 was positively correlated with the total writing score, as well as the content, organization, and language scores. Meanwhile, regarding interaction with the language partner, a change in difficulty was positively correlated with the content score; however, this was negatively related to a change in confidence.

In contrast to previous qualitative studies on the use of LLMs, such as ChatGPT, to support the L2 writing process (X. Li et al., 2023; Mah et al., 2024; M. Zou & Huang, 2024), the present study aimed to assess whether the writing scores following interaction with GPT-4 would equal or even surpass those obtained following interaction with a human language partner during the pre-writing phase. This was achieved through a within-subject behavioral experiment. M. Zou and Huang’s (2024) thematic analysis of interviews with doctoral students suggested that ChatGPT could support writers at the pre-writing stage. Although this finding appears to be partially inconsistent with the current study’s results, it should be noted that, in our study, the interaction with GPT-4 was limited to 10 min in the pre-writing phase. This is a relatively short preparation period and might have been insufficient in enabling GPT-4 to completely fulfill its function (cf., for instance, Shi et al., 2025, in which the participants underwent an 11-week interaction with ChatGPT, which promoted their writing performance). On the other hand, M. Zou and Huang (2024) examined the participants’ self-reflections, rather than directly comparing their writing scores. This is in line with our rating results, which indicated that the participants felt more familiar with the topics and confident in their writing, as well as experiencing lower levels of difficulty during the writing process, after interacting with GPT-4. Nevertheless, a change in writing confidence as rated by the participants could not guarantee a qualitative change in their writing performance, according to the current findings.

In addition, through further analysis of the interaction transcripts, it was found that notable differences existed between the language partner and GPT-4 in terms of personalized feedback, emotional support, and interaction modes. This was partially consistent with previous research (Eryılmaz & Başal, 2024; S. Zou et al., 2024). According to Vygotsky’s (1978) sociocultural theory, language acquisition is a social process that requires interaction and collaboration with more experienced individuals to complete tasks within the zone of proximal development (ZPD). Therefore, the native Chinese language partner, as an experienced individual, utilized heuristic questions such as “Could you tell me your general idea now?” to offer personalized feedback to the participating Chinese L2 learners. In addition, they implemented dynamic adjustments and provided context-aware affirmations such as “Okay, that’s great” to optimize the learners’ language and organization in the pre-writing phase, thereby enhancing their writing scores. Conversely, while GPT-4 is capable of generating linguistically accurate responses, it lacks the ability to engage in truly social and contextually adaptive interactions. These limitations may hinder GPT-4’s effectiveness in supporting Chinese L2 learners’ writing within their ZPD. Moreover, with regard to language and organization, GPT-4’s use of overly formal language (e.g., “Let’s define the generation gap”) and rigid structural frameworks (e.g., “Step 1: Thesis Statement → Step 2: Evidence Synthesis”) may result in the diversion of L2 learners from the core task of idea generation. According to the writing process model (Hayes & Flower, 1980), learners tend to focus on idea generation in the pre-writing stage. Therefore, GPT-4’s prescriptive approach may partly explain its limited effectiveness in supporting Chinese L2 learners’ writing performance as compared with the language partner, particularly in terms of language and organization.

Thus, our results support the notion that feedback from L2 language partners (or teachers) plays an essential role in L2 writing, as also suggested by Dong (2024), G. Kurt and Y. Kurt (2024), and G. L. Liu et al. (2024). Moreover, although studies utilizing qualitative approaches have claimed that LLMs help to improve L2 writing (Tam, 2025; G. Kurt & Y. Kurt, 2024), their findings are based on L2 learners’ subjective judgments, and they lack a systematic comparison with an orthogonal control group. Thus, such studies might overstate the contributions of ChatGPT-like models to L2 writing. Our results highlight that, at least during the short pre-writing phase, GPT-4 fails to elicit a significant improvement in writing scores, whereas interaction with a human language partner results in a prominent change that emerges rapidly.

Nonetheless, this study still highlights the potential benefits of LLMs in supporting L2 writing. Interaction with GPT-4 served to increase the participants’ writing confidence by providing more detailed information and improving their familiarity with the topics, decreasing the level of writing difficulty. As indicated by Agustini (2023), LLMs may promote autonomy in L2 learning, helping learners to self-control their learning processes and thus increasing their learning confidence. Moreover, in some aspects, interaction with GPT-4 could be as beneficial as interacting with a language partner. It is commonly believed that lowering the affective filter threshold is critical for successful L2 learning (Krashen, 1981; X. Zhang, 2023). Furthermore, it seems that “familiarity” is a more important feature in interacting with GPT-4, because a change in familiarity was significantly correlated with the total writing score and the scores for most sub-dimensions. This might be initially ascribed to the fact that LLMs can provide more detailed and comprehensive information related to the studied topics. Notwithstanding the mass of information collected by the LLM, there were no significant differences in participants’ topic familiarity between the two interactive modes (i.e., interaction either with GPT-4 or with the language partner). An alternative explanation could be that the participants treated GPT-4 as a tool for information searching to increase their familiarity with the topics (Shoufan, 2023); therefore, the mechanism by which LLMs are used to support L2 writing might be qualitatively different from that applied when interacting with a human language partner. While such tools could exert an overall effect on the total score and on almost all sub-dimensions, they are apparently insufficient to significantly improve L2 writing scores. In contrast, when interacting with a language partner, learners might show more empathy and treat the language partner as their mentor. As a consequence, the content, which is the core dimension of L2 writing, might become the key aspect that the participants discuss with this mentor. Thus, in our study, the perceived writing difficulty decreased, which was more *specific* to the content score. Interestingly, the participants were found to be overconfident after interacting with the language partner, leading to a decrease in their content scores. However, whether different attitudes towards LLMs—or, more precisely, the distinct underlying mechanisms of their use—could influence L2 writing outcomes remains to be examined in future studies.

Taken together, the findings of this study emphasize the complementary role of LLMs alongside traditional human-mediated interactions in language learning. However, several limitations should be acknowledged. Firstly, this study primarily employed a within-subject design. Although the interaction sessions were separated by an interval of at least 10 days, and we assumed that it would be difficult to transfer the interaction experiences from the previous session to the next one due to the change in the writing topics, carryover effects might not have been completely eliminated. A between-subject design might be necessary, although the individual differences between the groups should be carefully controlled. Future research is encouraged to explore between-subject differences to provide a more comprehensive understanding of the differences between different interaction modes. Secondly, this study only examined the correlations between topic familiarity, writing confidence, perceived difficulty, and the four dimensions of writing scores. Future research could further investigate these relationships by building more complex and hybrid statistical models (e.g., using structural equation modeling) and taking more factors into account to better understand the underlying cognitive mechanisms. Finally, this study focused exclusively on the pre-writing phase. Future studies could extend the investigation to the holistic writing phase to provide a more complete understanding of the role of LLMs and human language partners across the entire writing process.

## 6. Conclusions

The current study highlights the differential impacts of interactive scenarios involving GPT-4 and a human language partner in the L2 pre-writing phase. Our findings indicated that while both modes of interaction could enhance learners’ topic familiarity and writing confidence and reduce the tasks’ perceived level of difficulty, interaction with a human language partner within a limited pre-writing preparation period led to a more significant improvement in the total writing score, particularly in the organizational and linguistic dimensions. In contrast, a relatively short interaction with GPT-4 did not translate into substantial improvements in the participants’ writing scores within the limited preparation period before actual writing. Furthermore, this research underscores the role that LLMs such as GPT-4 may play in supporting L2 writing in the pre-writing phase. The positive correlation between increased topic familiarity—gained through GPT-4 interaction—and writing performance suggests its potential as a supportive tool, fostering familiarity and reducing the perceived difficulty in L2 learning contexts. A longer and more intensive interaction with LLMs might be beneficial in improving L2 learners’ writing quality, but this remains to be studied in the future.

## Data Availability

Data will be made available upon reasonable request.

## Supplementary Materials

The following supporting information can be downloaded at: https://www.mdpi.com/article/10.3390/bs15040540/s1.

## Appendix A

**Table A1:** Comparison of interaction transcripts.

Dimension of Comparison	Language Partner	GPT-4
Topic Introduction	Heuristic introduction: encourages students to think actively through specific questions.	Direct introduction: quickly asks core topic questions.
Content Support	Significantly personalized: adjusts content based on students’ background and specific questions.	Strong structure: provides a clear framework.
Emotional Support	Strong emotional resonance: increases students’ confidence with encouragement and affirmation.	More positive feedback: enhances confidence with encouraging phrases but lacks personalized interaction.
Language and Expression	Moderate complexity: adjusts expression difficulty based on students’ language levels.	Formal expression: uses more advanced written language, suitable for advanced learners.
Interaction Mode	Cooperative interaction: collaboratively discusses and forms content.	Supportive interaction: affirms students’ answers through feedback.

By systematically coding the records obtained from interactions with the language partner and GPT-4, distinct differences in their pedagogical approaches were identified. The language partner demonstrated personalized interaction through heuristic questioning (e.g., “Could you tell me your general idea now?”) and dynamic content adjustments tailored to individual learners’ cultural backgrounds or knowledge gaps. This approach prioritized collaborative engagement, fostering student-led exploration through an iterative dialog (e.g., “When we start writing later, what if we integrate X here?”), while enabling strong emotional resonance via context-aware affirmations (e.g., “Okay, that’s great”).

In contrast, GPT-4 emphasized structured content delivery through predefined frameworks (e.g., “Step 1: Thesis Statement → Step 2: Evidence Synthesis”) and efficient feedback and task alignment (e.g., “You can develop this topic from the following aspects.”). Its formal language expression (e.g., “Let’s conceive the ideas step by step.”) provided clarity for advanced learners but limited its adaptability to varying proficiency levels. While GPT-4 offered positive reinforcement (e.g., “Good effort! Refine the conclusion.”), its feedback lacked contextual awareness of learners’ emotional states or progress history.
