# Supplementary material for: Complementing but Not Replacing: Comparing the Impacts of GPT-4 and Native-Speaker Interaction on Chinese L2 Writing Outcomes
Source: Behav Sci (Basel). 2025 Apr 17;15(4):540. doi: 10.3390/bs15040540 (PMC12023996; doi:10.3390/bs15040540)
Supplement: Supplementary file 1 [file behavsci-15-00540-s001.zip › behavsci-3519989-supplementary.pdf]

Supplementary Materials

1. Descriptive statistics

Shapiro-Wilk tests were performed to assess whether the writing scores (Table S1-S3), rating scores (Table S4-S6), and the score differences (Table S7 & S8) follows a normal distribution. As can be seen from the results, several scores were not normally distributed, and thus non-parametric statistic methods were employed in the Main Text.

Table S1. Normality test results of the writing scores under the condition of "without interaction".

	totalW	contentW	organizationW	vocabularyW	languageW
Mean	78.913	24.072	17.029	18.913	18.899
Std. Deviation	2.987	0.853	0.619	1.097	0.837
Shapiro-Wilk	0.948	0.956	0.940	0.935	0.946
P-value of Shapiro-Wilk	0.268	0.388	0.184	0.137	0.239

Table S2. Normality test results of the writing scores under the condition of "interaction with GPT-4".

	totalG	contentG	organizationG	vocabularyG	languageG
Mean	79.420	23.986	17.159	19.101	19.174
Std. Deviation	2.327	0.735	0.437	0.819	0.643
Shapiro-Wilk	0.906	0.867	0.871	0.899	0.949
P-value of Shapiro-Wilk	0.033	0.006	0.007	0.024	0.285

Table S3. Normality test results of the writing scores under the condition of "interaction with the language partner".

	totalP	contentP	organizationP	vocabularyP	languageP
Mean	81.203	24.507	17.493	19.536	19.667
Std. Deviation	1.610	0.618	0.316	0.500	0.522
Shapiro-Wilk	0.960	0.934	0.874	0.947	0.892
P-value of Shapiro-Wilk	0.457	0.132	0.008	0.254	0.017

Table S4. Normality test results of the rating scores under the condition of "without interaction".

	familiarityW	difficultyW	confidenceW
Mean	2.826	3.478	3.174
Std. Deviation	1.114	0.665	0.650
Shapiro-Wilk	0.910	0.818	0.788
P-value of Shapiro-Wilk	0.042	< .001	< .001

Table S5. Normality test results of the rating scores under the condition of “interaction with GPT-4”.

	familiarityGpre	difficultyGpre	confidenceGpre	familiarityGpost	difficultyGpost	confidenceGpost
Mean	3.130	3.435	3.087	4.261	2.609	4.000
Std. Deviation	1.254	1.080	0.848	0.810	1.118	0.953
Shapiro-Wilk	0.869	0.893	0.840	0.787	0.918	0.831
P-value of Shapiro-Wilk	0.006	0.018	0.002	< .001	0.060	0.001

Table S6. Normality test results of the rating scores under the condition of “interaction with the language partner”.

	familiarityPpre	difficultyPpre	confidencePpre	familiarityPpost	difficultyPpost	confidencePpost
Mean	3.304	3.087	3.391	4.000	2.783	3.739
Std. Deviation	1.222	1.203	0.988	0.798	1.085	0.864
Shapiro-Wilk	0.877	0.872	0.883	0.842	0.852	0.861
P-value of Shapiro-Wilk	0.009	0.007	0.012	0.002	0.003	0.004

Table S7. Normality test results of the rating score differences under the condition of “interaction with GPT-4”.

	familiarityGpost_W	difficultyGpost_W	confidenceGpost_W	familiarityPpost_W	difficultyPpost_W	confidencePpost_W
Mean	1.435	−0.870	0.826	1.174	−0.696	0.565
Std. Deviation	1.472	1.180	0.887	1.267	1.295	0.896
Shapiro-Wilk	0.932	0.929	0.748	0.895	0.949	0.838
P-value of Shapiro-Wilk	0.121	0.102	< .001	0.020	0.281	0.002

Table S8. Normality test results of the rating score differences under the condition of “interaction with the language partner”.

	familiarityGpost_pre	difficultyGpost_pre	confidenceGpost_pre	familiarityPpost_pre	difficultyPpost_pre	confidencePpost_pre
Mean	1.130	−0.826	0.913	0.696	−0.304	0.348
Std. Deviation	1.217	1.267	0.848	0.974	1.020	0.885
Shapiro-Wilk	0.857	0.911	0.869	0.851	0.910	0.855
P-value of Shapiro-Wilk	0.004	0.043	0.006	0.003	0.041	0.003

2. Examples of ERNIE Bot’s Scoring Comments

Topic: “How to Solve the Generation Gap Problem”

Overall Analysis:
The article provides a detailed exploration of the causes and solutions to the generation gap problem. It is logically structured, with a clear theme and smooth expression. Through specific examples and explanations, the author offers readers a deeper understanding of the generation gap issue.

Dimension Evaluation:

1) **Content (30%)**:
Strengths: The content is comprehensive, covering the definition, causes, and solutions to the generation gap. The solutions section is particularly detailed, including suggestions such as improving communication and fostering mutual understanding.
Weaknesses: No significant weaknesses, but the article could further explore the uniqueness of generation gap issues in different family and cultural contexts.
Suggestions for Improvement: Include an analysis of generation gap problems in diverse family and cultural backgrounds to make the article more comprehensive.
Score: 24/30 (Content is complete and specific.)

2) **Organization (20%)**:
Strengths:The structure is compact, starting with the definition of the generation gap, followed by an exploration of its causes and solutions, and concluding with an emphasis on the key to resolving the issue.
Weaknesses: No significant weaknesses, but the conclusion could be more impactful, such as by adding a vision for the harmonious development of intergenerational relationships in the future.
Suggestions for Improvement: Enhance the conclusion with a forward-looking perspective or a call to action for harmonious intergenerational relationships.
Score:18/20 (Structure is compact but the conclusion could be strengthened.)

3) **Language** (25%):
Strengths: The language is fluent, with accurate word choice and clear, logical expression.
Weaknesses: No significant weaknesses, but the article could benefit from richer rhetorical devices to enhance its expressiveness.
Suggestions for Improvement: Incorporate rhetorical devices such as metaphors or personification to make the article more vivid and engaging.
Score: 22/25 (Language is fluent and accurate.)

4) **Vocabulary** (25%):
Strengths: The vocabulary is diverse and effectively conveys the theme and details.
Weaknesses: No significant weaknesses, but the article could introduce some professional terms or idioms to enhance its academic or cultural depth.
Suggestions for Improvement: Include professional terms or idioms, such as "intergenerational divide" or "seeking common ground while reserving differences."
Score: 22/25 (Vocabulary is diverse and accurate.)

Total Score: 86/100

3.Measurement of Teachers' Agreement Levels with ERNIE Bot's Scores

Three experienced Chinese teachers were required to read the 9 essays randomly selected under the interaction conditions (3 essays/condition), and rating the agreement levels in a 5-point Likert scale according to the ERNIE Bot's Scores and the corresponding rationales automatically provided by the ERNIE Bot.

The 5-Point Likert Scale:

Please evaluate the rationales of ERNIE Bot's scores across four dimensions (content, organization, language, and vocabulary) for each of the 9 essays based on your reading. For each dimension, the scoring range is 1-5 (1 = completely disagree, 2 = slightly disagree, 3 = neutral, 4 = comparatively agree, 5 = strongly agree). Thank you very much for your participation!

First Essay (as an example):

1. **Content**: What is your level of agreement with ERNIE Bot's score on the content dimension? (1-5 points)
    - 1 point: Completely disagree
    - 2 points: Slightly disagree
    - 3 points: Neutral
    - 4 points: Comparatively agree
    - 5 points: Strongly agree

2. **Organization**: What is your level of agreement with ERNIE Bot's score on the organization dimension? (1-5 points)
    - 1 point: Completely disagree
    - 2 points: Slightly disagree
    - 3 points: Neutral
    - 4 points: Comparatively agree
    - 5 points: Strongly agree

3. **Language**: What is your level of agreement with ERNIE Bot's score on the language dimension? (1-5 points)
    - 1 point: Completely disagree
    - 2 points: Slightly disagree
    - 3 points: Neutral
    - 4 points: Comparatively agree
    - 5 points: Strongly agree

4. **Vocabulary**: What is your level of agreement with ERNIE Bot's score on the vocabulary dimension? (1-5 points)
    - 1 point: Completely disagree
    - 2 points: Slightly disagree
    - 3 points: Neutral
    - 4 points: Comparatively agree
    - 5 points: Strongly agree

The averaged agreement rating scores of the three experienced Chinese teachers for each interaction condition could be found in Table S9.

Table S9. Teachers' Agreement Ratings with ERNIE Bot's Scores.

**Interactive Conditions**	**Dimension**	**Mean Scores**
Without interaction	Content	4.55
	Organization	4.44
	Vocabulary	4.66
	Langauge	4.33
Interaction with GPT-4	Content	4.44
	Organization	4.44
	Vocabulary	4.41
	Langauge	4.22
Interaction with the language partner	Content	4.44
	Organization	4.33
	Vocabulary	4.33
	Langauge	4.55
